# Inhibition of CaMKII Does Not Attenuate Cardiac Hypertrophy in Mice with Dysfunctional Ryanodine Receptor

**DOI:** 10.1371/journal.pone.0104338

**Published:** 2014-08-05

**Authors:** Asima Chakraborty, Daniel A. Pasek, Tai-Qin Huang, Angela C. Gomez, Naohiro Yamaguchi, Mark E. Anderson, Gerhard Meissner

**Affiliations:** 1 Department of Biochemistry and Biophysics, University of North Carolina, Chapel Hill, NC, United States of America; 2 Department of Regenerative Medicine and Cell Biology, Medical University of South Carolina, Charleston, SC, United States of America; 3 Division of Cardiovascular Medicine, Departments of Internal Medicine, and Molecular Physiology and Biophysics, University of Iowa, Iowa City, IA, United States of America; Virginia Commonwealth University, United States of America

## Abstract

In cardiac muscle, the release of calcium ions from the sarcoplasmic reticulum through ryanodine receptor ion channels (RyR2s) leads to muscle contraction. RyR2 is negatively regulated by calmodulin (CaM) and by phosphorylation of Ca^2+^/CaM-dependent protein kinase II (CaMKII). Substitution of three amino acid residues in the CaM binding domain of RyR2 (RyR2-W3587A/L3591D/F3603A, RyR2^ADA^) impairs inhibition of RyR2 by CaM and results in cardiac hypertrophy and early death of mice carrying the RyR2^ADA^ mutation. To test the cellular function of CaMKII in cardiac hypertrophy, mutant mice were crossed with mice expressing the CaMKII inhibitory AC3-I peptide or the control AC3-C peptide in the myocardium. Inhibition of CaMKII by AC3-I modestly reduced CaMKII-dependent phosphorylation of RyR2 at Ser-2815 and markedly reduced CaMKII-dependent phosphorylation of SERCA2a regulatory subunit phospholamban at Thr-17. However the average life span and heart-to-body weight ratio of *Ryr2^ADA/ADA^* mice expressing the inhibitory peptide were not altered compared to control mice. In *Ryr2^ADA/ADA^* homozygous mice, AC3-I did not alter cardiac morphology, enhance cardiac function, improve sarcoplasmic reticulum Ca^2+^ handling, or suppress the expression of genes implicated in cardiac remodeling. The results suggest that CaMKII was not required for the rapid development of cardiac hypertrophy in *Ryr2^ADA/ADA^* mice.

## Introduction

In cardiac muscle, excitation-contraction coupling in response to an action potential initiates an influx of Ca^2+^ ions via dihydropyridine-sensitive L-type Ca^2+^ channels (Ca_v_1.2). This triggers the massive release of Ca^2+^ from an intracellular Ca^2+^-storage organelle, the sarcoplasmic reticulum (SR), by opening type 2 ryanodine receptor ion channels (RyR2s) [1]. The released Ca^2+^ causes muscle contraction. Sequestration of released Ca^2+^ back into the SR by an ATP-dependent Ca^2+^ pump (SERCA2a) leads to muscle relaxation.

Ca^2+^/calmodulin-dependent protein kinase II (CaMKII) regulates the cellular entry of activator Ca^2+^ through Ca_v_1.2 and thereby SR Ca^2+^ release via RyR2 [1]–[4]. Phosphorylation of SERCA2a regulatory protein phospholamban (PLN) at Ser-16 by protein kinase A and Thr-17 by CaMKII enhances SR Ca^2+^ sequestration [5]. Site directed mutagenesis of the predominant CaMKII phosphorylation site of RyR2 to mimic constitutively phosphorylated (RyR2-S2815D) and dephosphorylated (S2815A) channels, showed that CaMKII-dependent phosphorylation of RyR2 increases channel open probability and the risk of heart failure in mice following transverse aortic constriction [6], [7].

Cardiac myocytes express two major CaMKII isoforms, γ and δ. Of these, CaMKIIδ has two splice variants, B and C. CaMKIIδ_B_ has a nuclear localization signal and transcriptionally regulates signaling pathways in cardiac myopathies [8]–[10]. Overexpression of CaMKIIδ_B_ or CaMKIIδ_C_ induced transactivation of myocyte enhancer factor 2 (MEF2)-dependent gene expression and up-regulation of hypertrophic marker genes [11]. Overexpression of cytosolic CaMKIIδ_C_ increased RyR2 and PLN phosphorylation, enhanced Ca^2+^ spark activity, and reduced SR Ca^2+^ content [11], [12]. CaMKIIδ knockout mice had no major changes in ventricular structure and function [13], [14]. However, after pressure overload induced by transaortic banding surgery, cardiac remodeling was reduced in CaMKIIδ deficient mice, which exhibited inhibition of RyR2 phosphorylation and reduced SR Ca^2+^ leak [13], [14]. The results suggested that inhibition of CaMKII may limit the development of heart failure.

Based on the understanding of CaMKII as a pathological signaling molecule in cardiomyopathies, we asked whether an active strategy of chronic myocardial-targeted CaMKII inhibition could prevent or reduce cardiac hypertrophy in a mouse model (*Ryr2^ADA/ADA^* mice) with a well-defined mutation in RyR2. *Ryr2^ADA/ADA^* mice have three substituted amino acid residues in the calmodulin (CaM) binding domain of RyR2 (RyR2-W3587A/L3591D/F3603A, RyR2^ADA^) that disrupt its CaM inhibition at diastolic and systolic Ca^2+^ concentrations and result in cardiac hypertrophy and the early death of *RyR2^ADA/ADA^* mice [15]. While wild-type and *Ryr2^ADA/ADA^* mice had comparable CaMKII activities in 1-day old mice using an *in vitro* kinase assay [15], these studies did not rule out an *in vivo* procardiomyopathic role of CaMKII in *Ryr2^ADA/ADA^* mice. Additionally, *in vitro* measurements of CaMKII activity do not necessarily reflect the cellular activities in mice. Differences in Ca^2+^ handling due to CaM impairment of RyR2 function and CaM distribution due to loss of RyR2 CaM binding may result in altered CaMKII activity in homozygous mutant hearts, which are difficult to assess in an *in vitro* assay.

To determine whether CaMKII inhibition could prevent or reduce cardiac hypertrophy, we crossed mutant mice with mice transgenically expressing CaMKII autocamtide 3 inhibitory peptide (AC3-I) or control peptide (AC3-C). Transgenic overexpression of AC3-I protected mouse hearts against pathological remodeling in response to myocardial infarction and β-adrenergic stimulation [16]. The present study shows that CaMKII inhibitory peptide AC3-I reduced phosphorylation of PLN at Thr-17 in *Ryr2^+/+^* and *Ryr2^ADA/ADA^* mice without significantly altering life span, cardiac morphology and performance, or markers of cardiac hypertrophy relative to mice expressing the control peptide. The findings suggest that the pathological effects of the RyR2^ADA^ mutation are independent of myocardial CaMKII.

## Materials and Methods

### Ethics Statement

This study was carried out in accordance with the recommendations in the Guide for the Care and Use of Laboratory Animals of the National Institutes of Health. The protocol was approved by the University of North Carolina at Chapel Hill Institutional Animal Care and Use Committee (10-062).

### Materials

[^3^H]Ryanodine was obtained from Perkin Elmer Life Sciences. Protease and phosphatase inhibitor cocktails were from Sigma. Rabbit polyclonal antibody F9221 against RyR2 amino acid sequence 1372–1387 was produced by New England Peptide. Rabbit polyclonal antibody pRyR2 on Ser-2809 (A010-30AP) was from Badrilla (Leeds, UK). Rabbit polyclonal antibody to pRyR2 on Ser-2815 was the generous gift of Dr. Andrew Marks. Mouse monoclonal antibody PLN (A010-14) and rabbit polyclonal antibodies pPLN on Ser-16 (A010-12) and Thr-17 (A010-13) were from Badrilla (Leeds, UK). Rabbit polyclonal antibody pCaMKII on Thr-286 (ab32678) and two rabbit polyclonal antibodies against the conserved N-terminal (ab136400) and C-terminal (ab37999) regions of CaMKIIγ and CaMKIIδ were from Abcam. Comparable results were obtained using the two CaMKII antibodies. Rabbit polyclonal antibodies protein kinase D (PKD) (#2052) and pPKD on S744/8 (#2054) and S916 (#2051) were from Cell Signaling Technology. Chemicals were from Sigma-Aldrich unless specified otherwise.

### Genetically modified mice

*Ryr2^+/ADA^* mice [15] were mated with mice expressing the CaMKII inhibitory AC3-I peptide or the control AC3-C peptide [16]. Mice with AC3-I or AC3-C peptide expression were each backcrossed at least 5 times to 129Svev genetic background. Nine different genotypes of mice were obtained by crossing *Ryr2^+/ADA^* mice expressing either of the two peptides according to Mendelian Law. Of these, the 4 genotypes investigated were *Ryr2^+/+^* mice expressing AC3-I or AC3-C, and *Ryr2^ADA/ADA^* mice expressing AC3-I or AC3-C.

### Echocardiography

To determine left ventricular cardiac function, transthoracic M-mode echocardiography was performed on restrained, unanesthetized 10-day old mice, using the Vevo 2100 high resolution imaging system (VisualSonics) with 40 MHz probe [15]. Mice were restrained by taping down gently on a warmed board (Indus Industries for VisualSonics).

### Morphological analysis

Hearts from 9–11 day old mice were fixed with 4% (w/v) paraformaldehyde in PBS (pH 7.4) and dehydrated using increasing concentrations of ethanol in water [15]. Paraffin embedded hearts were sectioned to 6–9 µm thickness and stained as described [15].

### Quantitative RT-PCR

Gene expression was measured by quantitative RT-PCR using the ABI Prism 7700 Sequence Detector (Applied Biosystems) [17]. RNA was isolated from left ventricles of 10-day-old mice using the ABI Prism 6700 Automated Nucleic Acid Workstation according to the manufacturer's protocol. Primers and fluorogenic probes for β-myosin heavy chain (β-MHC), atrial natriuretic peptide (ANP) and brain natriuretic peptide (BNP) were described [18]. Levels of gene expression as a percentage of *Ryr2^+/+^/AC3-C* were determined relative to β-actin.

### Preparation of heart homogenates

Hearts of 10-day old mice were homogenized using a Tekmar Tissumizer for 3×7 s at a setting of 13,500 rpm in 20 mM imidazole, pH 7.0, 0.3 M sucrose, 0.15 M NaCl, protease and phosphatase inhibitor cocktails, 25 mM β-glycerophosphate, 5 mM NaF and 2.5 mM NaVO_4_. Homogenates were stored in small aliquots at −80°C. Protein concentrations were determined using BCA assay.

### Immunoblot *analyses*

Homogenates (20 µg protein/lane) were separated by SDS/PAGE and transferred to nitrocellulose membranes [19]. Membranes were blotted with 2% Advance blocking reagent (Amersham Biosciences) in 0.5% Tween 20, Tris buffered saline (TBS), pH 7.4 at 24°C and probed with primary antibodies and secondary peroxidase-conjugated IgG antibody. Immunoblots were developed using enhanced chemiluminescence and quantified using ImageQuantTL Analysis Software.

### [^3^H]Ryanodine binding

Specific binding of [^3^H]ryanodine to RyRs was measured to determine the number of RyR high affinity binding sites [20]. Cardiac muscle homogenates were incubated for 4–5 h at 24°C with a near saturating concentration of 20 nM [^3^H]ryanodine in 20 mM imidazole, pH 7.0, 0.6 M KCl, 0.1 mM Ca^2+^, and protease inhibitors as described [15]. Nonspecific binding was determined using 1000-fold excess of unlabeled ryanodine.

### ^45^Ca^2+^ uptake rate

ATP-dependent ^45^Ca^2+^ uptake rates by homogenates were determined using a filtration assay as described [15]. ^45^Ca^2+^ uptake rates were determined in presence of 6 µM KN93, a CaMKII specific inhibitor.

### Data analysis

Results are expressed as mean ± SEM. Differences between samples were analyzed using one or two way ANOVA followed by Tukey test.

## Results

**Figure 1:**
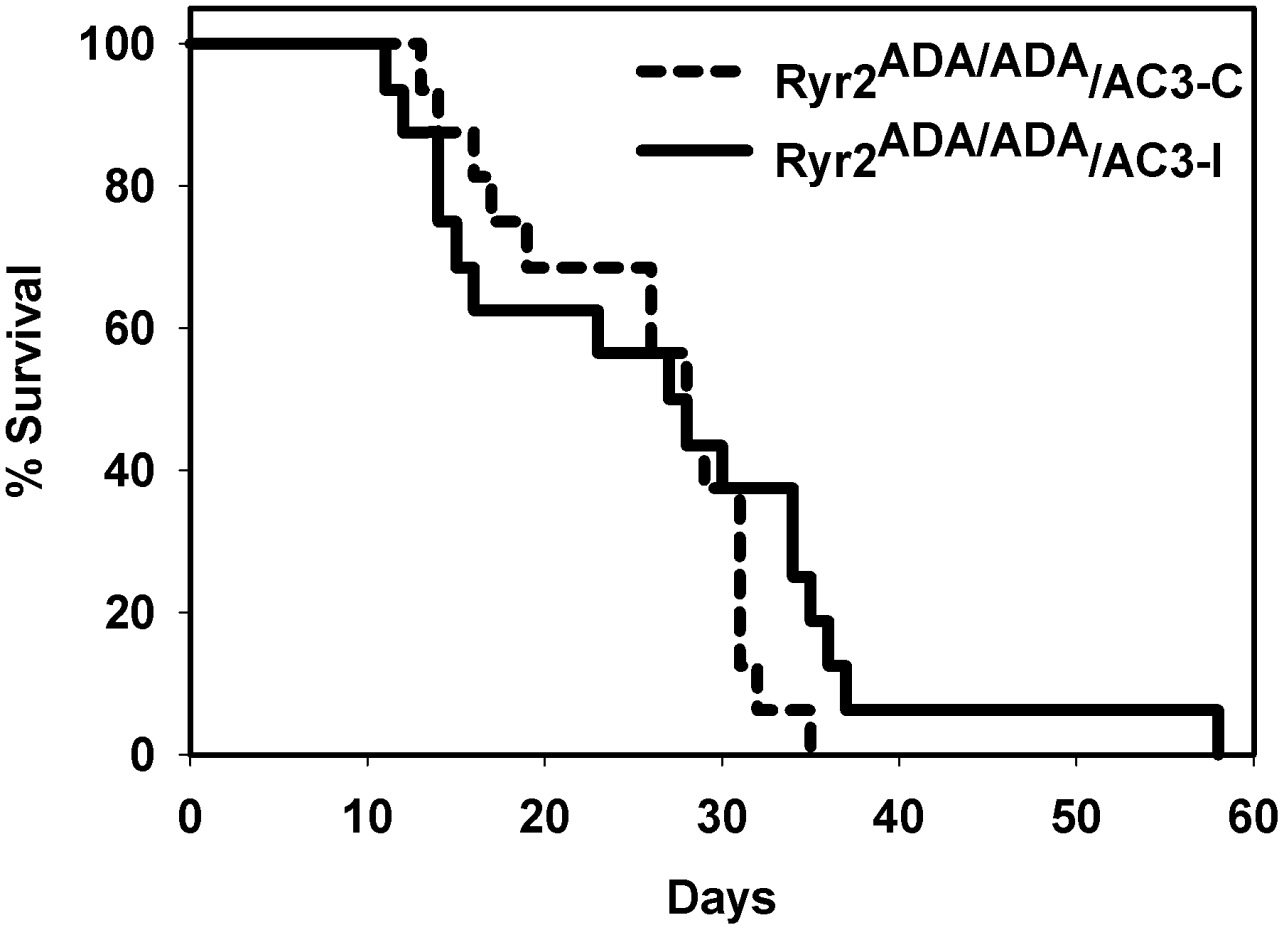
Survival of *Ryr2 ^ADA/ADA^/AC3-C* and *Ryr2^ADA/ADA^/AC3-I* mice. Mean lifetimes ± SEM of *Ryr2^ADA/ADA^* mice expressing CaMKII control AC3-C and inhibitory AC3-I peptides were 26.4±1.6 (n = 16) and 28.1±3.0 (n = 16) days, respectively.

*Ryr2^+/ADA^* mutant mice expressing the CaMKII inhibitory peptide AC3-I or control peptide AC3-C were mated to obtain *Ryr2^+/+^* and *Ryr2^ADA/ADA^* mice expressing AC3-I or AC3-C. Sixteen *Ryr2^ADA/ADA^* mice expressing AC3-I died 12 to 58 days after birth compared with 16 mutant mice expressing the control peptide that died 14 to 35 days after birth (Fig. 1). Mean lifetimes were 28.1±3.0 and 26.4±1.6 days for *Ryr2^ADA/ADA^* mice expressing the inhibitory and control peptides, respectively. Thus, no marked difference in life span occurred between the two groups.

**Figure 2:**
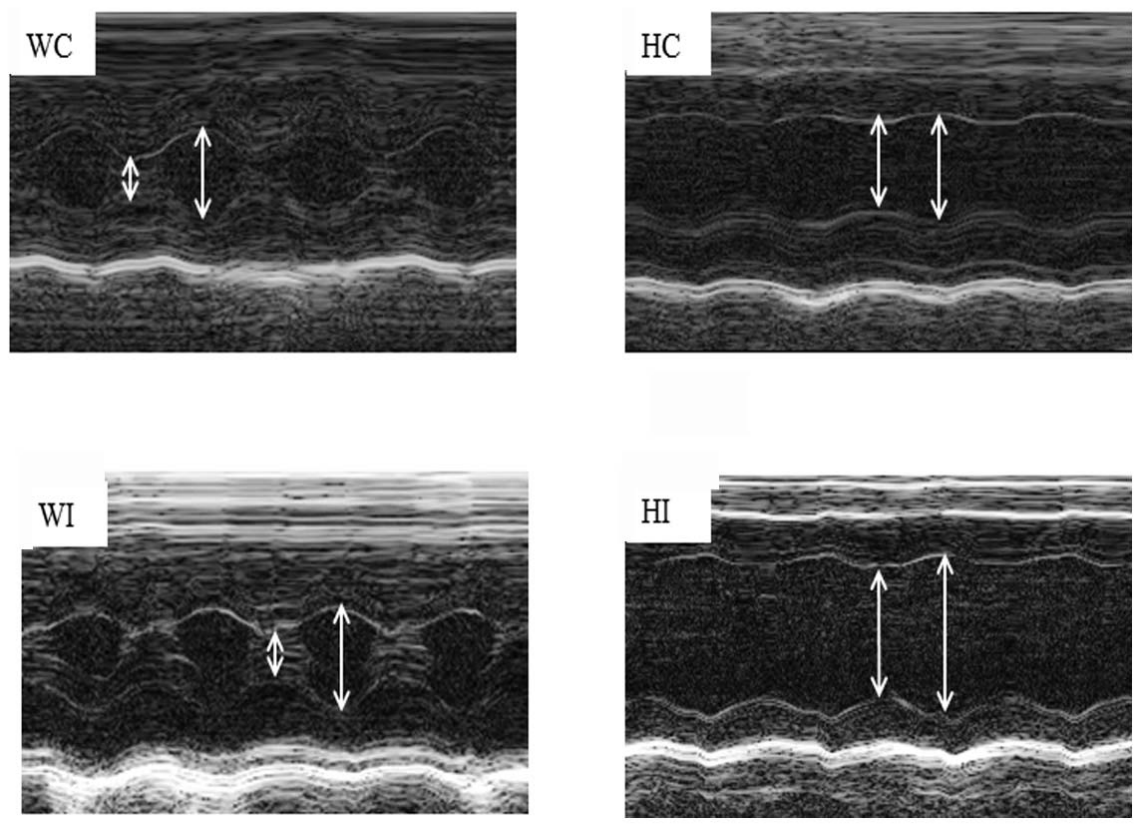
Echocardiograms of wild-type and mutant hearts. Representative M-mode echocardiograms of 10-day old *Ryr2^+/+^/AC3-C* (WC) and/*AC3-I* (WI) and *Ryr2^ADA/ADA^/AC3-C* (HC) and/*AC3-I* (HI) mice are shown. Left ventricular end-diastolic (right arrows) and end-systolic (left arrows) dimensions are indicated.

The effects of expressing AC3-I and AC3-C in *Ryr*2^+/+^ and *Ryr2^ADA/ADA^* mice were examined further at day 10 after birth. Expression of the AC3-I inhibitory peptide did not suppress an increase in left ventricular weight-to-body weight ratio of *Ryr2^ADA/ADA^* mice compared with mutant mice expressing the AC3-C control peptide (Table 1). Left ventricle function of 10-day old *Ryr2^+/+^* and *Ryr2^ADA/ADA^* mice expressing AC3-I or AC3-C was compared by conscious M-mode echocardiography. Heart rates of *Ryr2^ADA/ADA^* mice expressing AC3-I or AC3-C peptide of 477 and 484 beats per min (bpm) were significantly slower than the 612 and 613 bpm for *Ryr2^+/+^* mice expressing either of the two peptides (Table 1). Left ventricular end-diastolic (LVEDD) and end-systolic dimensions (LVESD) were significantly increased in *Ryr2^ADA/ADA^* mice compared with *Ryr2^+/+^* mice in the absence of the peptides (Fig. 2, Table 1). Fractional shortening (FS) calculated from the two above parameters and ejection fraction (EF) were significantly reduced in mutant mice. However, there was no significant difference between wild-type or mutant mice expressing AC3-C or AC3-I (Table 1). The data suggest that inhibition of CaMKII by AC3-I did not improve cardiac performance of *Ryr2^ADA/ADA^* mice compared to mutant mice expressing the control peptide.

**Table 1:** Echocardiography of 10-day old mice double targeted for RyR2^ADA^ and AC3 peptides.

Parameters	*Ryr2^+^* ^/+^	*Ryr2^+^* ^/+^	*Ryr2^ADA/ADA^*	*Ryr2^ADA/ADA^*
	*AC3-C*	*AC3-I*	*AC3-C*	*AC3-I*
	(n = 8)	(n = 6)	(n = 8)	(n = 6)
LV/BW	0.142±0.010	0.153±0.016	0.267±0.034^a^	0.312±0.042^a^
HR (bpm)	612±26	613±20	484±14^a^	477±18^a^
LVEDD (mm)	1.63±0.10	1.58±0.10	3.01±0.27^a^	2.93±0.19^a^
LVESD (mm)	0.59±0.07	0.65±0.11	2.52±0.29^a^	2.38±0.21^a^
FS (%)	64.3±3.4	59.4±6.1	17.5±2.2^a^	19.3±1.9^a^
IVSD (mm)	0.84±0.07	0.89±0.09	0.80±0.04	0.70±0.04
IVSS (mm)	1.29±0.07	1.29±0.13	1.00±0.05^a^	0.90±0.05^a^
LVPWD (mm)	0.70±0.06	0.72±0.05	0.94±0.12	0.78±0.07
LVPWS (mm)	1.08±0.07	1.09±0.07	1.17±0.11	0.99±0.09
EF(%)	92.8±2.4	89.9±2.1	38.1±4.3^a^	44.1±2.1^a^

LV/BV, left ventricular weight to body weight; HR, heart rate; bpm, beats/min; LVEDD, left ventricular end diastolic dimension; LVESD, left ventricular end systolic dimension; FS, fractional shortening (LVEDD - LVESD)/LVEDD; IVSD, interventricular septum diastolic thickness; IVSS, interventricular septum systolic thickness; LVPWD, left ventricular posterior wall diastolic thickness; LVPWS, left ventricular posterior wall systolic thickness, EF, ejection fraction. Data are the mean ± SEM of number of mice shown in parenthesis.

a p<0.05 compared to *Ryr2^+/+^/AC3-C* and *Ryr2^+/+^/AC3-I* using one way ANOVA.

**Figure 3:**
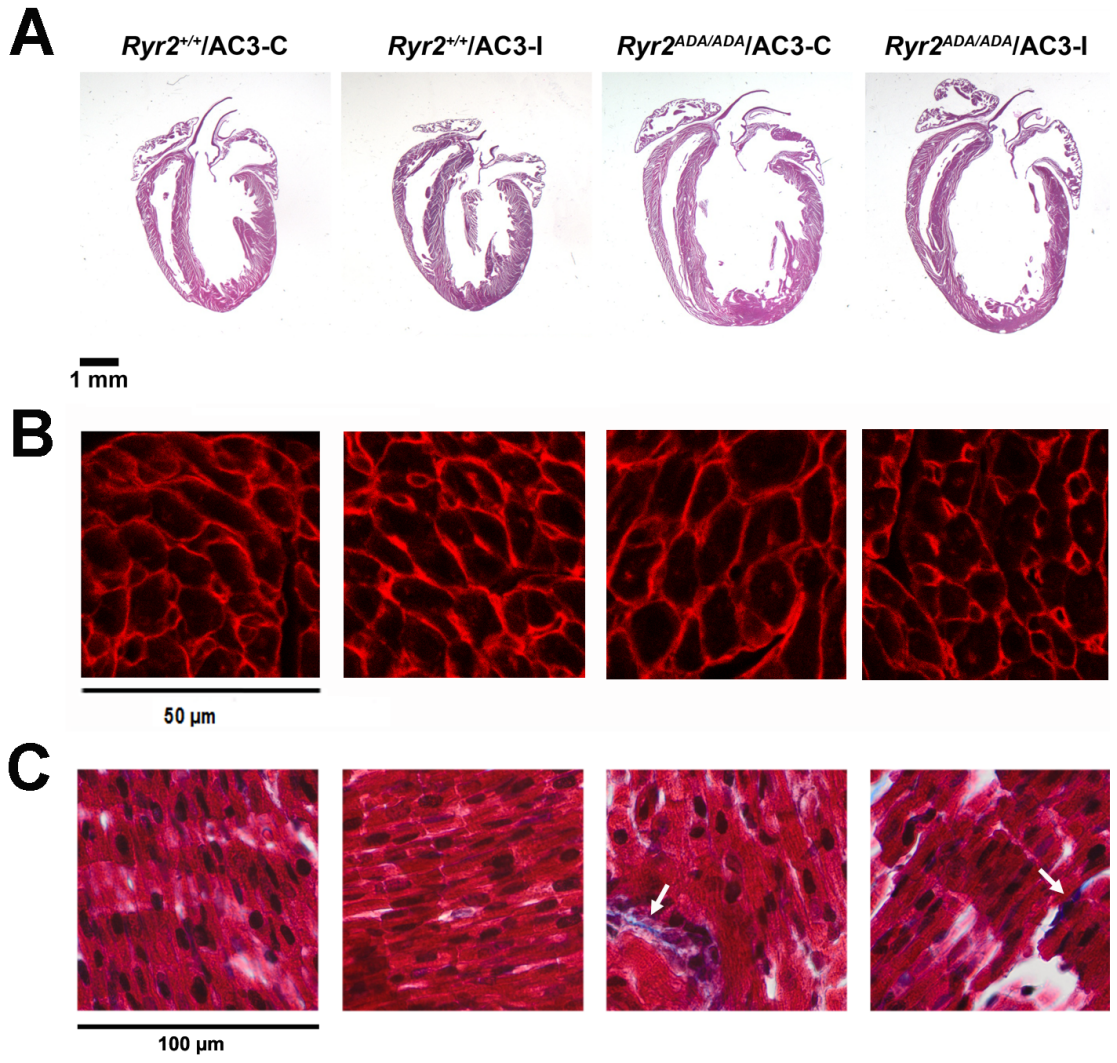
Morphological analysis of wild type and mutant hearts. (A) Representative sections of hearts from the indicated mice stained with hematoxylin and eosin. (B) Left ventricle sections were stained with TRITC-conjugated wheat germ agglutinin. Cross-sectional areas (µm^2^, n = 10 for each heart) were (in parentheses) for 2 *Ryr2^+/+^/AC3-C* (67.9±2.3), 3 *Ryr2^+/+^/AC3-I* (66.8±2.8), 4 *Ryr2^ADA/ADA^/AC3-C* (96.0±3.0) and 4 *Ryr2^ADA/ADA^/AC3-I* (86.9±3.3) hearts. The significantly increased cross-sectional area in *Ryr2^ADA/ADA^* hearts compared to *Ryr2^+/+^* hearts was not significantly altered by AC3-I compared to AC3-C using one way ANOVA. (C) Papillary muscle stained with Masson Trichrome. Arrows indicate collagen deposits.

Morphological analysis confirmed the generation of hypertrophied hearts and dilated left ventricle chambers in *Ryr2^ADA/ADA^* mice with control or inhibitory peptides (Fig. 3A). Consistent with left ventricle/body weight ratio (Table 1), there were no noticeable differences in heart size between AC3-C and AC3-I groups. Heart sections from 5 wild-type and 8 mutant mice expressing either AC3-C or AC3-I were stained with TRITC-conjugated wheat germ agglutinin and cellular cross-sectional areas were determined. Cross-sectional areas in *Ryr2^ADA/ADA^* hearts were significantly increased compared to *Ryr2^+/+^* hearts (Fig. 3B). However, there was no significant difference between wild-type or mutant mice expressing AC3-C or AC3-I. Masson Trichrome staining indicated negligible collagen deposition in the papillary muscle of wild-type mice. *Ryr2^ADA/ADA^* mice showed an occasional appearance of collagen (Fig. 3C, arrows). However, no obvious differences were observed between *Ryr2^+/+^* or *Ryr2^ADA/ADA^* mice expressing AC3-C or AC3-I.

*Ryr2^ADA/ADA^* mice are germline knock-in mice. While changes in other tissues cannot be ruled out, the RyR2 mutation appeared to primarily affect the heart, because body weight ratios were unchanged for lung, liver and brain.

**Figure 4:**
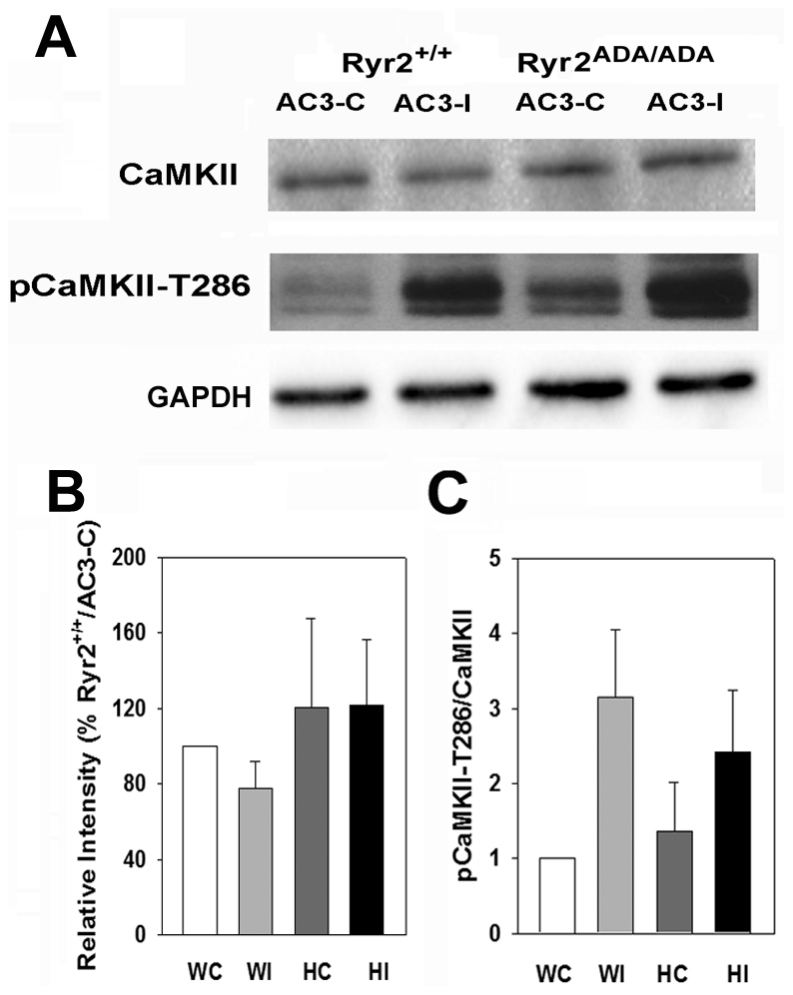
Expression of CaMKII and pCaMKII-T286 in heart homogenates. (A) Immunoblots of CaMKII and pCaMKII-T286 of heart homogenates from 10-day old *Ryr2^+/+^/AC3-C* (WC) and/*AC3-I* (WI) and *Ryr2^ADA/ADA^/AC3-C* (HC) and/*AC3-I* (HI) mice. Glyceraldehyde-3 phosphate dehydrogenase was the loading control. (B) Protein levels of CaMKII normalized to *Ryr2^+/+^/AC3-C*. (C) pCaMKII/CaMKII on Thr-286. Data were obtained analyzing proteins from 4–6 hearts of each genotype and are the mean ± SEM of 15–16 (B) and 5–7 (C) determinations using two way ANOVA. None of the differences were significant.

Immunoblot analysis showed that similar CaMKII protein levels were present in heart homogenates of wild-type and mutant mice expressing AC3-C or AC3-I peptides (Figs. 4A and B). We also determined autophosphorylation of CaMKII on Thr-286, which switches the kinase from a Ca^2+^/CaM-dependent to Ca^2+^/CaM-independent state. In agreement with a previous study [16], autophosphorylation of CaMKII increased (not significant) in wild-type and mutant mice expressing AC3-I compared to mice expressing AC3-C (Figs. 4A and C). However, increased pCaMKII-T286/CaMKII ratio was not expected to enhance CaMKII activity in AC3-I mice, because autophosphorylated CaMKII is inhibited by AC3-I [16].

**Figure 5:**
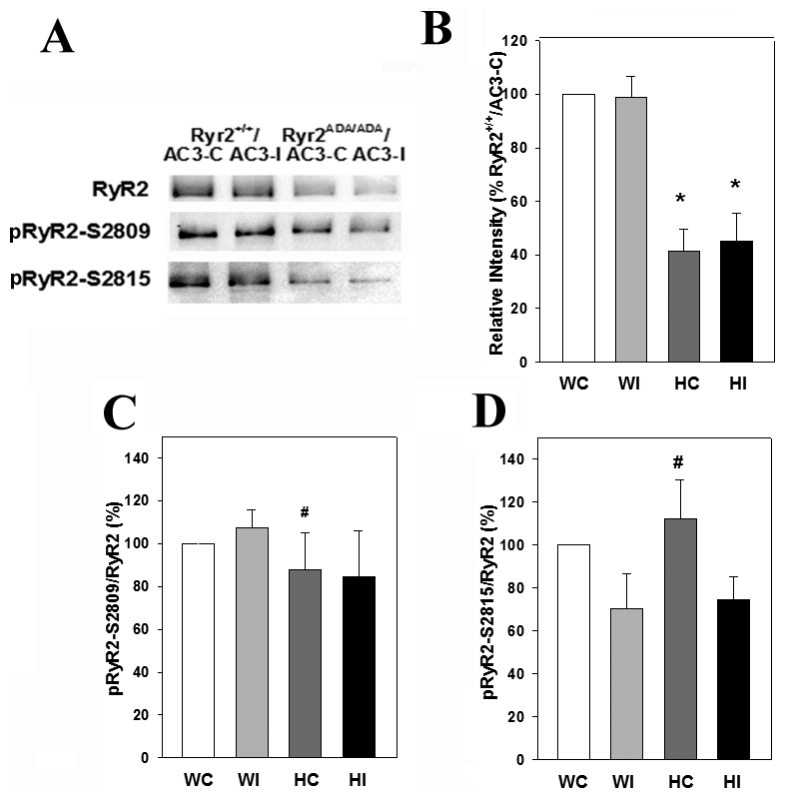
RyR2 phosphorylation on Ser-2809 and Ser-2815 in heart homogenates. (A) Immunoblots of RyR2, pRyR2-S2809 and pRyR2-S2815 of heart homogenates from 10-day old *Ryr2^+/+^/AC3-C* (WC) and/*AC3-I* (WI) and *Ryr2^ADA/ADA^/AC3-C* (HC) and/*AC3-I* (HI) mice. Equal amounts of proteins (20 µg) were loaded on gels. (B) Intensity of RyR2 protein bands normalized for *Ryr2^+/+^/AC3-C* protein band intensities. (C and D) pRyR2/RyR2 on S2809 and S2815, respectively. Data were obtained analyzing proteins from 4–6 hearts of each genotype and are the mean ± SEM of 13 (B), 4 (C) and 5–7 (D) determinations using two way ANOVA. *p<0.05 compared with WC and WI. ^#^p<0.05 compared with HC in blot of total RyR2 shown in B.

To verify that AC3-I inhibited CaMKII activity in AC3-I mice, phosphorylation levels of RyR2 and phospholamban (PLN) were determined by immunoblot analysis. RyR2 is phosphorylated at Ser-2809 by protein kinase A (PKA) and at Ser-2815 by CaMKII [21], [22]. In agreement with previous studies [15], total RyR2 protein expression was reduced by ∼60% in homozygous mice compared to WT expressing inhibitory and control AC3 peptides (Fig. 5). In hearts expressing the control peptide, increased pRyR2-S2809/RyR2 and pRyR2-S2815/RyR2 ratios were observed in mutant hearts compared with total RyR2 protein. AC3-I reduced (not significant) pRyR2-S2815/RyR2 phosphorylation ratios in wild-type and mutant hearts compared with hearts expressing the control peptide. One caveat is that we could not distinguish between mice carrying transgenes of AC3-I and AC3-C in one allele or both. It is therefore conceivable that in mice expressing fewer copies, the AC3-I concentration was suboptimal in inhibiting CaMKII associated with RyR2 [22].

**Figure 6:**
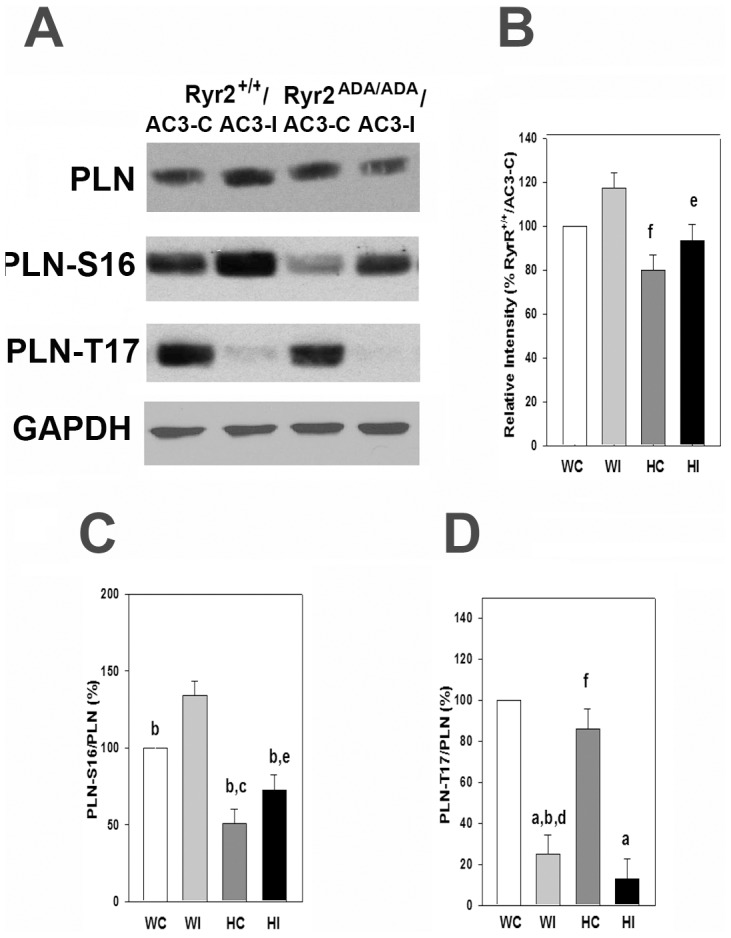
PLN phosphorylation on Ser-16 and Thr-17 in heart homogenates. (A) Immunoblots of PLN, pPLN-Ser16 and pPLN-Thr17 of heart homogenates from 10-day old *Ryr2^+/+^/AC3-C* (WC) and/*AC3-I* (WI) and *Ryr2^ADA/ADA^/AC3-C* (HC) and/*AC3-I* (HI) mice. Glyceraldehyde-3 phosphate dehydrogenase was the loading control. (B) Intensity of PLN protein bands normalized for *Ryr2^+/+^/AC3-C* protein band intensities. (C and D) pPLN/PLN on Ser-16 and Thr-17, respectively. Data were obtained analyzing proteins from 4–6 hearts of each genotype and are the mean ± SEM of 19–22 (B), 12 (C) and 11–12 (D) determinations using two way ANOVA. ^a^p<0.05 compared to WC-T17 and HC-T17, ^b^p<0.05 compared to WI-S16, ^c^p<0.05 compared to WC-S16, ^d^p<0.05 compared to WI-PLN, ^e^p<0.05 compared to HI-T17 ^f^p<0.05 compared to HC-S16.

PLN has two physiologically relevant phosphorylation sites, Ser-16 phosphorylated by PKA and Thr-17 phosphorylated by CaMKII [5]. Immunoblots in Fig. 6 show similar total PLN protein levels in *Ryr2^+/+^* and *Ryr2^ADA/ADA^* mice harboring AC3-C or AC3-I peptides. Among PLN, pPLN-S16/PLN and pPLN-T17/PLN panels a number of significant changes were observed. Comparable pPLN-T17/PLN phosphorylation ratios were present in *Ryr2^+/+^* and *Ryr2^ADA/ADA^* hearts expressing the control peptide. In contrast, phosphorylation ratios of pPLN-T17/PLN decreased by 75% in *Ryr2^+/+^* hearts compared to an 85% decrease in *Ryr2^ADA/ADA^* hearts expressing the inhibitory peptide. This suggests that AC3-I inhibited CaMKII activity in *Ryr2^+/+^* and *Ryr2^ADA/ADA^* hearts.

**Figure 7:**
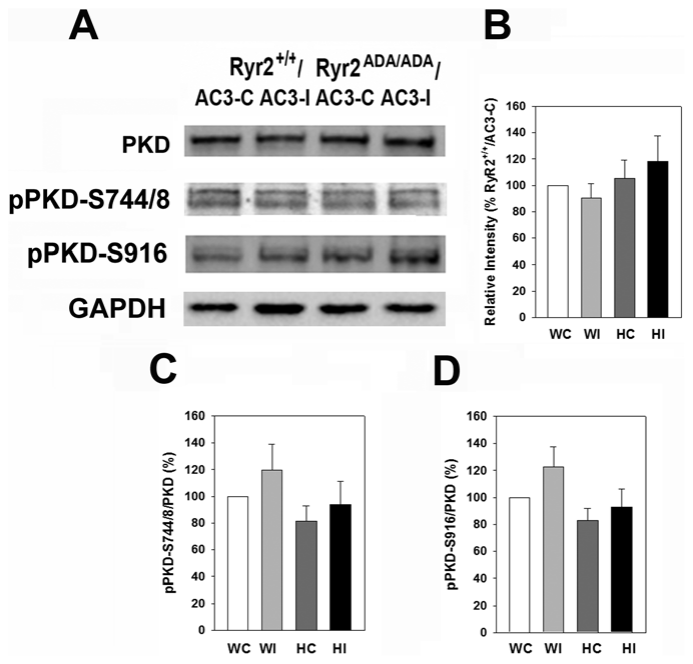
Expression of PKD, pPKD-S744/S748 and pPKD-S916 in heart homogenates. (A) Immunoblots of PKD, pPKD-S744/748 and pPKD-S916 of heart homogenates from 10-day old *Ryr2^+/+^/AC3-C* (WC) and/*AC3-I* (WI) and *Ryr2^ADA/ADA^/AC3-C* (HC) and/*AC3-I* (HI) mice. Glyceraldehyde-3 phosphate dehydrogenase was the loading control. (*B*) Intensity of protein PKD bands in *RyR2^+/+^* and *RyR2^ADA/ADA^* hearts normalized for RyR2^+/+^/AC3-C protein band intensities. (C and D) pPKD/PKD on S744/748 and S916, respectively. Data were obtained analyzing proteins from 4–5 hearts of each genotype and are the mean ± SEM of 13 (B), 13(C) and 12 (D) determinations using two way ANOVA. None of the differences were significant.

Protein kinase D (PKD) is a member of the CaMK superfamily [23] and has been reported to be inhibited by AC3-I [14]. Fig. 7 compares protein levels of PKD and phosphorylation of PKD on Ser-744/Ser-748 and Ser-916 in wild-type and mutant hearts expressing AC3-I or AC3-C peptides. Similar PKD protein levels (Fig. 7B) and pPKD/PKD phosphorylation ratios (Figs. 7C and D) were observed in hearts of wild-type and *Ryr2^ADA/ADA^* mice.

**Figure 8:**
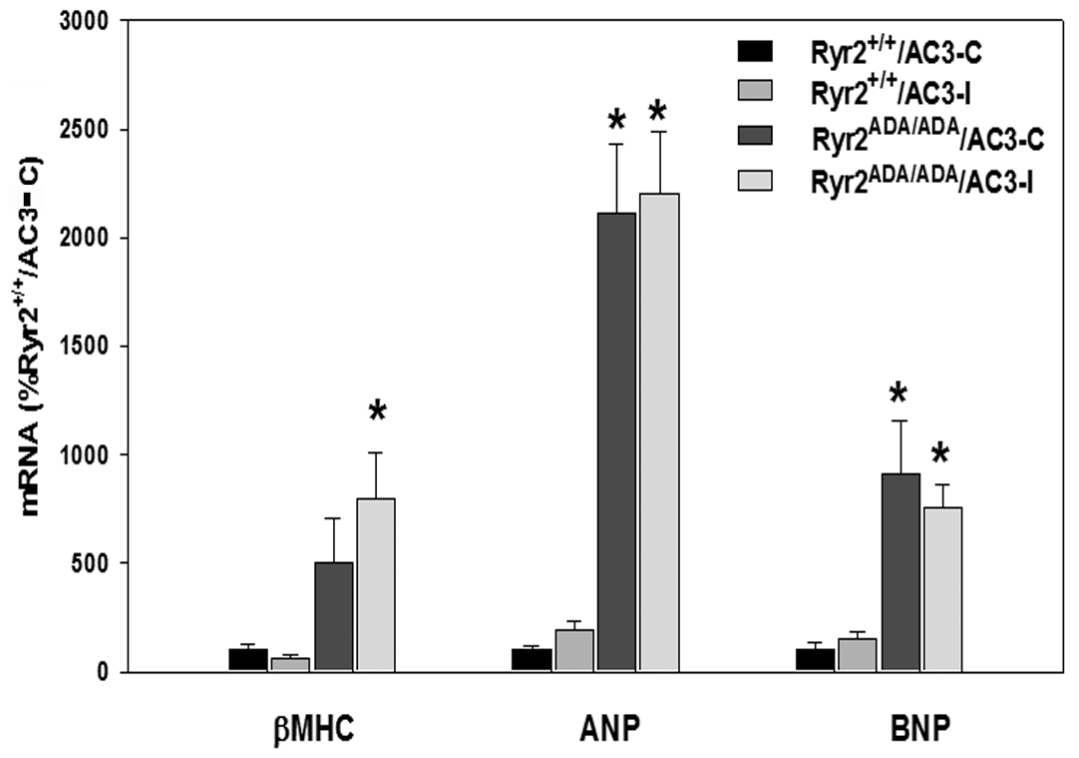
Quantitative RT-PCR of mRNA from mice double-targeted for RyR2^ADA^ and AC3 peptides. RT-PCR analyses were performed using total RNA from the left ventricle of 10-day old mice. β-MHC, β-myosin heavy chain; ANP, atrial natriuretic peptide; BNP, brain natriuretic peptide. Data are the mean of 7–8 left ventricle samples using one way ANOVA. p<0.05 compared with *Ryr2^+/+^/AC3-C*.

We tested whether AC3-I altered the expression of several genes linked to cardiac hypertrophy. In agreement with our previous report [15], quantitative RT-PCR showed that mRNA levels of β-MHC, ANP and BNP increased in the left ventricle of 10-day old *Ryr2^ADA/ADA^* mice compared to *Ryr2^+/+^* mice (Fig. 8). There were no marked differences in the expression levels of these genes in *Ryr2^+/+^* mice expressing AC3-I or AC3-C. Similarly, the expression levels of β-MHC, ANP and BNP were not significantly altered in *Ryr2^ADA/ADA^* mice expressing AC3-I or AC3-C.

**Figure 9:**
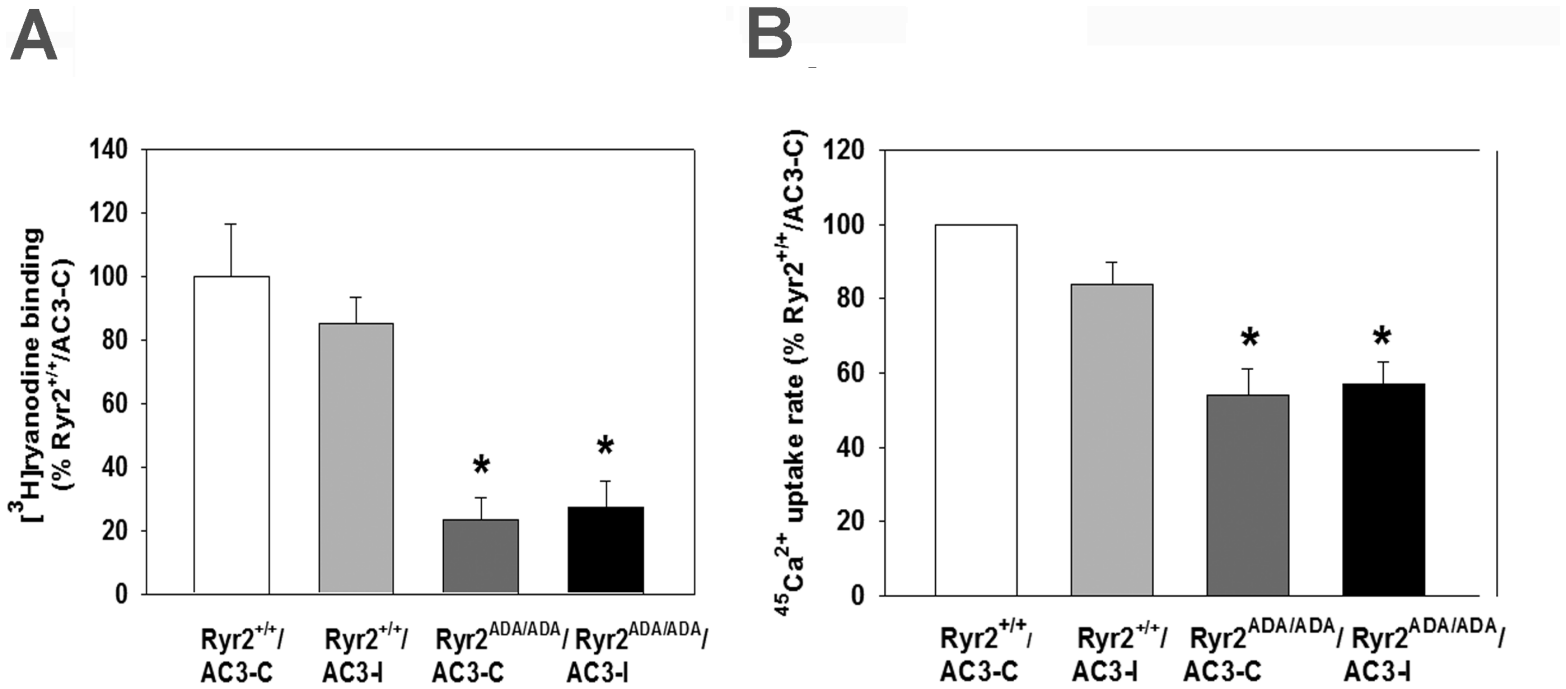
B_max_ of [^3^H]ryanodine binding (A) and ^45^Ca^2+^ uptake rates (B) of heart homogenates from 10-day-old mice double-targeted for RyR2^ADA^ and AC3 peptides. Data were obtained analyzing homogenates from 4 hearts of each genotype and are the mean ± SEM of 4 (A) and 6–7 (B) determinations using one way ANOVA. *p<0.05 compared with *Ryr2^+/+^/AC3-C* (A, B) and/*AC3-I* (A).

We then asked whether AC3-I altered SR Ca^2+^ handling of *Ryr2^+/+^* and *Ryr2^ADA/ADA^* mice. B_max_ values of [^3^H]ryanodine binding, which are a measure of RyR2 protein levels, and ^45^Ca^2+^ uptake rates, which reflect SR Ca^2+^ pump activity, were determined. The significantly reduced B_max_ of [^3^H]ryanodine binding in *Ryr2^ADA/ADA^* hearts compared to *Ryr2^+/+^* [15] was not altered further by AC3-I or AC3-C in *Ryr2^+/+^* and *Ryr2^ADA/ADA^* mice (Fig. 9A). In mutant mice, reduced ^45^Ca^2+^ uptake rates (Fig. 9B) were likely due to decreased SERCA2a protein content. In heart homogenates of the mutant mice, we measured decreased SERCA2a protein content (not shown); however, corresponding experiments with AC3 homogenates have not been done. ^45^Ca^2+^ uptake rates were reduced to a level similar in *Ryr2^ADA/ADA^* hearts expressing AC3-C or AC3-I compared to *Ryr2^+/+^* hearts expressing AC3-I or AC3-C peptides. SR Ca^2+^ uptake was likely not reduced by AC3-I because PLN-S16 phosphorylation, which was less affected by AC3-I compared to PLN-17 (Fig. 6), was sufficient to mediate a maximal β-agonist-mediated cardiac response in perfused hearts [24]. To compensate for the lower AC3-I concentration due to sample dilution, experiments were done in the presence of CaMKII inhibitor KN93. Taken together, the results suggest that the CaMKII inhibitory peptide did not improve depressed cardiac Ca^2+^ handling of *Ryr2^ADA/ADA^* mice.

## Discussion

CaMKII and CaM modulate cardiac muscle function by regulating multiple ion channels and transport systems [2]. We previously showed the physiological importance of an interaction between RyR2 and CaM in cardiac muscle by generating mice expressing mutant RyR2 (RyR2^ADA^) with disrupted CaM regulation [15]. *Ryr2^ADA/ADA^* mice develop cardiac hypertrophy by postnatal day 1 and die within 2 weeks after birth. Impaired CaM regulation of RyR2 resulted in upregulation of ERK/p90RSK signaling and reduced GSK-3β activity in E16.5 heart homogenates [19]. Calcineurin A-β and class II HDAC/MEF2 signaling pathways, which have a critical role in pathological hypertrophy regulated by CaMKII [25], were up-regulated in 1-day and 10-day old but not E16.5 Ryr2^ADA/ADA^ hearts [19]. In the present study, pRyR2-S2815 phosphorylation was not increased in the mutant mice. The result suggests that CaMKII-mediated RyR2 phosphorylation does not have an essential role in the *Ryr2^ADA/ADA^* phenotype.

Heart failure is a major public health problem without adequate therapies. Since CaMKII has emerged as a procardiomyopathic signal [2], [26]–[31], our goal was to determine whether CaMKII inhibition has a protective role in a severe form of cardiomyopathy due to impairment of CaM inhibition of RyR2. The effect of specific inhibition of RyR2 phosphorylation by CaMKII was shown by deleting the CaMKII S2815 phosphorylation site of RyR2. Genetic ablation of RyR2-S2815 phosphorylation indicated that mutant mice were more resistant than wild-type mice against pacing-induced arrhythmias following transverse aortic constriction [6], development of heart failure [7], and arrhythmias following reperfusion of hearts [32].

We mated *Ryr2^+/ADA^* mice with transgenic mice that expressed CaMKII inhibitory peptide AC3-I or the inactive control peptide AC3-C. AC3-I protected against structural heart disease by mimicking the conserved sequence of the CaMKII regulatory domain [16]. These mouse models also provided evidence that CaMKII regulates atrioventricular nodal conduction [33]. Inhibition of CaMKII by AC3-I reduced the up-regulation of proinflammatory genes [34] and protected against apoptosis [35] during myocardial infarction. In an *in vitro* kinase assay, we found that wild-type and *Ryr2^ADA/ADA^* mice had comparable CaMKII activity [15]. This previous study was performed using 1-day old RyR2^ADA/ADA^ homogenates at a very early stage-of-onset of cardiac hypertrophy. Cardiac hypertrophy rapidly progresses in *Ryr2^ADA/ADA^* mice, becoming severe by day 10 leading to the early death of the mutant mice. The present *in vivo* study that addresses the role of CaMKII in late stage severe cardiac hypertrophy of 10-day old mutant mice substantiated earlier *in vitro* results using 1-day homogenates. CaMKII phosphorylated RyR2 to a similar extent in wild-type and mutant hearts in late stage severe cardiac hypertrophy. Inhibition of CaMKII did not significantly alter the development of cardiac hypertrophy of *Ryr2^ADA/ADA^* mice. CaMKII inhibition resulted in a significant reduction of PLN phosphorylation at Thr-17 in *Ryr2^+/+^* and *Ryr2^ADA/ADA^* hearts expressing AC3-I.

We generated and characterized a second mutant mouse model with a single amino acid substitution (L3591D, RyR2^D^) in the CaM binding domain [36]. In contrast to *Ryr2^ADA/ADA^* mice, *Ryr2^D/D^* mice showed loss of CaM inhibition at diastolic but not systolic Ca^2+^, had a normal lifespan, and showed only modest changes in heart size and function. This suggested that loss of CaM regulation in *Ryr2^D/D^* mice does not have a major impact as in *Ryr2^ADA/ADA^* mice, implying that CaM inhibition of RyR2 at systolic Ca^2+^ is necessary for normal cardiac growth and function [36]. This was an unexpected finding because a “leaky” RyR2 at diastolic Ca^2+^, caused by phosphorylation of RyR2 at Ser2815, has been implicated in cardiomyopathies [29], [31], [37]. Our results suggest that CaMKII did not significantly alter the development of cardiac hypertrophy of *Ryr2^ADA/ADA^* mice at either diastolic or systolic Ca^2+^.

Protein kinase D (PKD), a member of the CaMK superfamily [23], has been reported to be inhibited by AC3-I [14]. While a direct interaction with RyR2 has not been established, PKD, like CaMKII, phosphorylates class II histone deacetylases, which triggers their nuclear export, thereby regulating transcription and promoting cardiac remodeling [14], [38], [39]. Immunoblots did not show significant differences in total PKD, pPKD-S744/S748 and pPKD-S916 protein levels between 10-day old wild-type and mutant mice expressing AC3 control or inhibitory peptides (Fig. 7). However, phosphorylation at these sites does not necessarily reflect PDK activity in mutant and wild-type hearts. PKD binds diacylglycerol, which is formed by a variety of cellular stimuli, inducing cellular redistribution of the enzyme, and potentially altering PKD activity [23], [40]. Therefore, physiological relevance of *in vitro* measurements is uncertain, using the isolated form of the enzyme. To our knowledge there are no unique targets for PKD that could have been used to assess *in vivo* PKD activity.

In conclusion, the results suggest that CaMKII does not have an essential role in the rapid development of cardiac hypertrophy and early death of mutant mice impaired in CaM regulation of RyR2 at diastolic and systolic Ca^2+^ concentrations. Although RyR2 mutations that impair CaM regulation in cardiac pathologies have not yet been identified, our studies should help to understand the role of RyR2 regulation by CaM and CaMKII in cardiac hypertrophy and heart failure in humans.
